# What Will It Take to Eliminate Pediatric HIV? Reaching WHO Target Rates of Mother-to-Child HIV Transmission in Zimbabwe: A Model-Based Analysis

**DOI:** 10.1371/journal.pmed.1001156

**Published:** 2012-01-10

**Authors:** Andrea L. Ciaranello, Freddy Perez, Jo Keatinge, Ji-Eun Park, Barbara Engelsmann, Matthews Maruva, Rochelle P. Walensky, Francois Dabis, Jennifer Chu, Asinath Rusibamayila, Angela Mushavi, Kenneth A. Freedberg

**Affiliations:** 1 Division of Infectious Disease, Medical Practice Evaluation Center, Massachusetts General Hospital, Boston, Massachusetts, United States of America; 2 INSERM U897 Africa Team of the Institut de Santé Publique, d'Epidémiologie et de Développement, Université Bordeaux Segalen, Bordeaux, France; 3 HIV/AIDS Unit, Pan American Health Organization, Washington, District of Columbia, United States of America; 4 Zimbabwe Country Office, Elizabeth Glaser Pediatric AIDS Foundation, Harare, Zimbabwe; 5 Division of General Medicine, Medical Practice Evaluation Center, Massachusetts General Hospital, Boston, Massachusetts, United States of America; 6 Organization of Public Health Interventions and Development, Harare, Zimbabwe; 7 Division of Infectious Disease, Brigham and Women's Hospital, Boston, Massachusetts, United States of America; 8 Ministry of Health and Child Welfare, Harare, Zimbabwe; 9 Department of Health Policy and Management, Harvard School of Public Health, Boston, Massachusetts, United States of America; 10 Harvard University Center for AIDS Research, Boston, Massachusetts, United States of America; Rwanda Ministry of Health, Rwanda

## Abstract

**Background:**

The World Health Organization (WHO) has called for the “virtual elimination” of pediatric HIV: a mother-to-child HIV transmission (MTCT) risk of less than 5%. We investigated uptake of prevention of MTCT (PMTCT) services, infant feeding recommendations, and specific drug regimens necessary to achieve this goal in Zimbabwe.

**Methods and Findings:**

We used a computer model to simulate a cohort of HIV-infected, pregnant/breastfeeding women (mean age, 24 y; mean CD4, 451/µl; breastfeeding duration, 12 mo). Three PMTCT regimens were evaluated: (1) single-dose nevirapine (sdNVP), (2) WHO 2010 guidelines' “Option A” (zidovudine in pregnancy, infant nevirapine throughout breastfeeding for women without advanced disease, lifelong combination antiretroviral therapy for women with advanced disease), and (3) WHO “Option B” (pregnancy/breastfeeding-limited combination antiretroviral drug regimens without advanced disease; lifelong antiretroviral therapy with advanced disease). We examined four levels of PMTCT uptake (proportion of pregnant women accessing and adhering to PMTCT services): reported rates in 2008 and 2009 (36% and 56%, respectively) and target goals in 2008 and 2009 (80% and 95%, respectively). The primary model outcome was MTCT risk at weaning.

The 2008 sdNVP-based National PMTCT Program led to a projected 12-mo MTCT risk of 20.3%. Improved uptake in 2009 reduced projected risk to 18.0%. If sdNVP were replaced by more effective regimens, with 2009 (56%) uptake, estimated MTCT risk would be 14.4% (Option A) or 13.4% (Option B). Even with 95% uptake of Option A or B, projected transmission risks (6.1%–7.7%) would exceed the WHO goal of less than 5%. Only if the lowest published transmission risks were used for each drug regimen, or breastfeeding duration were shortened, would MTCT risks at 95% uptake fall below 5%.

**Conclusions:**

Implementation of the WHO PMTCT guidelines must be accompanied by efforts to improve access to PMTCT services, retain women in care, and support medication adherence throughout pregnancy and breastfeeding, to approach the “virtual elimination” of pediatric HIV in Zimbabwe.

*Please see later in the article for the Editors' Summary*

## Abstract 2

Using a simulation model, Andrea Ciaranello and colleagues find that the latest WHO PMTCT (prevention of mother to child transmission of HIV) guidelines plus better access to PMTCT programs, better retention of women in care, and better adherence to drugs are needed to eliminate pediatric HIV in Zimbabwe.

## Editors' Summary

**Background:**

A woman who is infected with HIV can pass the virus to her baby during pregnancy, labor and delivery, or breastfeeding—mother-to-child HIV transmission (MTCT). Without treatment, up to 30% of babies born to HIV-infected women will become infected with HIV during pregnancy or at delivery, and a further 5%–20% will become infected through breastfeeding. In 2009, around 400,000 children under 15 years of age became infected with HIV, mainly through MTCT—90% of these MTCT infections occurred in Africa.

In addition to preventing HIV infection among prospective parents and avoiding unwanted pregnancies among HIV-positive women, effective prevention of MTCT (PMTCT) requires preventing the transmission of HIV from infected mothers to their infants during pregnancy, labor, delivery, and breastfeeding.

In 2010, the World Health Organization (WHO) published new guidelines for PMTCT based on combination antiretroviral therapy for women with advanced HIV disease, and two options for countries to select for women with less advanced disease. Option A includes zidovudine (ZDV) during pregnancy and single-dose nevirapine (sdNVP) at delivery, followed by daily nevirapine syrup for infants throughout the duration of breastfeeding; Option B includes maternal triple-drug ARV regimens throughout pregnancy and breastfeeding. However, WHO estimates that only 53% of pregnant women worldwide received any antiretroviral medicines for PMTCT in 2009.

**Why Was This Study Done?:**

As in many sub-Saharan African countries where prolonged breastfeeding is common, and necessary to improve child health, Zimbabwe is implementing the 2010 WHO guidelines with Option A. However, because of the challenges of enrolling and retaining women in PMTCT programs, the effectiveness of this strategy is unknown. Therefore in this study, the researchers used a model to calculate the level of PMTCT uptake in Zimbabwe, the PMTCT drug regimens, and the duration of breastfeeding that would be necessary to reach the WHO goal of an MTCT risk below 5%.

**What Did the Researchers Do and Find?:**

The researchers used a validated computer simulation model developed for analyzing the cost-effectiveness of preventing AIDS complications to measure risk of infant HIV transmission at the time of weaning, the HIV infection risk at 4–6 weeks of age, infant survival at two years of age, and 2-year HIV-free survival. The researchers used four scenarios of PMTCT uptake and linked the models to two populations of pregnant and breastfeeding women (mean age, 24 years) in Zimbabwe, and then analyzed the combinations of the factors necessary to reach MTCT risks less than 5%.

At baseline, the researchers found that the 2008 National PMTCT Program in Zimbabwe led to a projected 12-month MTCT risk of 20.3%. The projected risk in 2009 was 18.0% because of improved uptake. The estimated MTCT risk with Option A at 56% uptake (2009 levels) was 14.4% and with Option B was 13.4%. However, even with greatly increased uptake, such as 95% levels, the researchers found that projected transmission risks would exceed the WHO goal of less than 5% MTCT, and that the MTCT risk would fall below 5% at the 95% uptake level only if the lowest transmission risks were used for each drug regimen, or if breastfeeding duration were shortened.

**What Do These Findings Mean?:**

These findings show that the planned implementation of the 2010 WHO PMTCT guidelines with Option A in Zimbabwe could substantially reduce infant HIV infection risk compared to the 2009 national program with sdNVP. Furthermore, in order to reach a MTCT risk of less than 5%, a national program based on either Option A or Option B will also need to include strategies to improve access to PMTCT services (to almost 100% uptake), retain women in care, and support medication adherence throughout pregnancy and breastfeeding. These findings from a resource-limited country with high HIV prevalence and prolonged breastfeeding may be useful for other countries in sub-Saharan Africa.

**Additional Information:**

Please access these websites via the online version of this summary at http://dx.doi.org/10.1371/journal.pmed.1001156.

- Avert gives some more information on MTCT and PMTCT.
- The United Nations Children's Fund has factsheets on national PMTCT responses in the most affected countries.
- WHO's strategic vision for PMTCT for 2010–2015 is also available.

## Introduction

When antiretroviral drugs (ARVs) are administered during pregnancy, and breastfeeding is avoided, mother-to-child HIV transmission (MTCT) may occur in fewer than 1% of pregnancies [1]. These highly effective prevention strategies have led the pediatric HIV epidemic to be nearly eliminated in the United States and Europe [1],[2]. In settings where breastfeeding is recommended for improved infant health [3], recent trials have demonstrated that the provision of ARVs to breastfeeding mothers or their infants can reduce total MTCT rates to 1%–5% at 6 mo of age [4]–[15]. Based on these encouraging results, in 2009, HIV prevention and treatment organizations emphasized a new goal: the “virtual elimination” of pediatric HIV infection, defined as the reduction of MTCT to less than 5% [16]–[20]. Toward this goal, the World Health Organization (WHO) released new guidelines for the prevention of MTCT (PMTCT) in 2010, based on combination antiretroviral therapy (ART) for women with advanced HIV disease, and country-level selection between two antiretroviral options during pregnancy and breastfeeding (“Option A” or “Option B”) for women with less advanced disease [21]. Option A includes zidovudine (ZDV) during pregnancy and single-dose nevirapine (sdNVP) at delivery, followed by daily nevirapine (NVP) syrup for infants throughout the duration of breastfeeding; Option B includes maternal triple-drug ARV regimens throughout pregnancy and breastfeeding (Table A in Text S1) [21].

**Figure 1:**
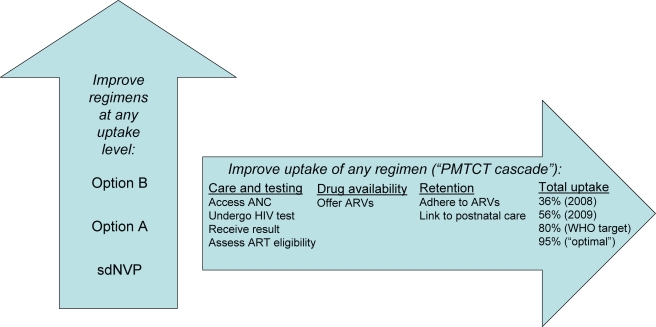
Two dimensions for potential improvements in PMTCT in Zimbabwe. This figure shows the “two dimensions” in which PMTCT services can be improved. First, along the vertical arrow, PMTCT programs can transition to more intensive drug regimens (i.e., from sdNVP to Option A to Option B). Second, along the horizontal arrow, programs can undertake interventions to improve “uptake” of PMTCT services, defined as the proportion of pregnant, HIV-infected women who receive and adhere to ARVs for PMTCT, for example, from 36% in 2008 to 56% in 2009, and perhaps to 80% or 95% with future scale-up effort. Within the horizontal arrow are depicted the three “domains” of uptake examined in these analyses: care and testing, drug availability, or retention. sdNVP represents the current National PMTCT Program, based largely on sdNVP; “Option A” and “Option B” are the WHO 2010 PMTCT guideline-recommended regimens, as defined in the text and Text S1.

However, opportunities for PMTCT may be lost at each step in a “cascade” of care, from presentation to antenatal care (ANC), through HIV testing and result receipt, to ARV adherence through the many months of pregnancy and breastfeeding (Figure 1; Figure A in Text S1 [22]–[25]. The WHO estimates that only 53% of pregnant women worldwide received any ARVs for PMTCT in 2009 [19]. As a result, more than 370,000 new perinatal HIV infections were estimated to have occurred in 2009; the majority of these were in sub-Saharan Africa, where access to pediatric HIV therapy is limited and infant mortality is extremely high [19],[26].

Since 2002, the Zimbabwean national PMTCT program has provided ART to pregnant women with clinical evidence of WHO stage 3/4 disease, and sdNVP to all others [27]. As in many sub-Saharan African countries where prolonged breastfeeding is common, Zimbabwe is implementing the 2010 WHO guidelines with Option A. Because of the challenges of enrolling and retaining women in care at each step in the PMTCT cascade, it is not known how effective this strategy will be. We used a simulation model to project the level of PMTCT uptake in Zimbabwe, the PMTCT drug regimens, and the duration of breastfeeding that would be necessary to reach the WHO “virtual elimination” goal of an MTCT risk below 5%.

## Methods

### Analytic Overview

We expanded a validated computer simulation model of MTCT [28],[29] to include each step in the “cascade” of PMTCT-related care, from first presentation at ANC through 2 y postpartum (Figure A in Text S1). The MTCT model simulates a single pregnancy, delivery, and postpartum period for each mother–infant pair, and projects infant outcomes at birth. Additional clinical events for mothers and infants through 2 y after delivery are simulated using the CEPAC (Cost-Effectiveness of Preventing AIDS Complications) model (Figure B in Text S1) [30]–[32]. The primary outcome of the linked CEPAC and MTCT models is risk of infant HIV transmission at the time of weaning; secondary outcomes include HIV infection risk at 4–6 wk of age, 2-y pediatric survival, and 2-y HIV-free pediatric survival.

This analysis examined improvements along two dimensions of PMTCT care: more effective PMTCT regimens, and improved uptake of the PMTCT “cascade” (Figure 1). We examined three possible PMTCT regimens in Zimbabwe: (1) the 2002–2009 National PMTCT Program, based on sdNVP alone, (2) WHO 2010 guidelines' Option A, and (3) WHO 2010 guidelines' Option B. Infant outcomes were projected at four levels of PMTCT uptake (36%, 56%, 90%, and 95% of women/infants receiving medications by the time of infant delivery) [19],[23]. Key sensitivity analyses examined the impacts of duration of breastfeeding, maternal HIV disease stage, the range of published MTCT risks for each PMTCT regimen, and a “full” (100%) uptake scenario.

### Population

The linked models were used to simulate two populations of pregnant and breastfeeding women in Zimbabwe, with mean age of 24 y (standard deviation [SD]: 5 y) [33]. Cohort 1 included women already HIV-infected at their first ANC visit (regardless of whether HIV status was known to patients or providers). Cohort 2 was composed of all women becoming pregnant each year in Zimbabwe, an estimated 392,460 women [34],[35], with HIV prevalence of 16% at first ANC visit and HIV incidence of 1%/year during late pregnancy and breastfeeding [36]. Cohort 1 was thus nested within Cohort 2. Cohort 1 was analyzed to project MTCT rates, and Cohort 2 to project the proportion and number of infants in an annual birth cohort anticipated to become HIV-infected by 18 mo of age. All women were assumed to breastfeed their infants for 12 mo in the base case, based on WHO infant feeding guidelines [3].

### PMTCT Regimens and PMTCT Uptake Scenarios

The antenatal, intrapartum, and postpartum/neonatal components of each modeled PMTCT regimen are detailed in Table A in Text S1. In all modeled regimens, women identified as eligible for ART (CD4≤350/µl or WHO stage 3/4 disease) were referred for ART. The availability of ART after referral depended on the modeled PMTCT uptake scenario; the availability of CD4 assays was varied in sensitivity analyses. Following convention, we refer to combination antiretroviral therapy regimens as “ART” when intended for treatment of maternal HIV disease (also effective for PMTCT) and as “triple-drug ARV regimens” when offered to non-ART-eligible women for PMTCT.

To create the four uptake scenarios, the “cascade” of PMTCT services was categorized into three broad domains (Table 1; Figure 1): “care and testing,” “drug availability,” and “retention.” We then calculated a “product of participation” (POP), defined as the product of the proportions of women/infants receiving care in each of these three domains, both at delivery and at the end of the breastfeeding period [23]. Four primary scenarios of PMTCT uptake were simulated for each PMTCT regimen (Table 1). The first two scenarios reflected WHO and Zimbabwean Ministry of Health and Child Welfare estimates of uptake at each step in the PMTCT cascade for Zimbabwe in 2008 (total POP at delivery: 36%) and 2009 (total POP at delivery: 56%) [19],[23],[36],[37]. The “WHO target” scenario modeled the current WHO goal that 80% of pregnant women be HIV-tested and 80% of HIV-infected women receive PMTCT services [20],[37]. The “optimal” uptake scenario simulated 95% uptake of the complete PMTCT cascade through delivery, likely representing the best practically achievable outcomes and lowest MTCT risks for the evaluated PMTCT regimens. A scenario of full, 100% uptake was also examined in sensitivity analyses (Tables D–G in Text S1), to represent the maximum potential biologic efficacy of each regimen.

**Table 1:** PMTCT uptake scenarios.

Scenario	Care and Testing^a^	Drug Availability^b^	Retention^c^	POP^d^
**Zimbabwe National PMTCT Program 2008** [23]	ANC: 94% [23]; HIV testing: 75% [37]; result return: 99% [36]; total: 70% ( = 94%×75%×99%)	86%	Before delivery: 60% (mean of [11],[24],[43]–[47])	To delivery: 36% [23]
			Postpartum: if ANC, 87% (mean of [11],[43],[44],[48]–[50]); if no ANC, 43% (assumption: 50%)	To 18 mo postpartum: 31%
**Zimbabwe National PMTCT Program 2009** [19]	ANC: 91% [36]; HIV testing: 87% [36]; result return: 99% [36]; total: 78% ( = 91%×87%×99%)	82%	Before delivery: 87% [43]	To delivery: 56% [19]
			Postpartum: if ANC, 87% (mean of [11],[43],); if no ANC, 43% (assumption: 50%)	To 18 mo postpartum: 49%
**WHO target** [37]	ANC: 100%; HIV testing: 80%; result return: 100%; total: 80% ( = 100%×80%×100%)	100%	Before delivery: 100%	To delivery: 80%
			Postpartum: if ANC, 87% (mean of [11],[43],[44],[48]–[50]); if no ANC, 43% (assumption: 50%)	To 18 mo postpartum: 70%
**Optimal (95%) uptake**	ANC: 100%; HIV testing: 95%; result return: 100%; total: 95% ( = 100%×95%×100%)	100%	Before delivery: 100%	To delivery: 95%
			Postpartum: if ANC, 87% (mean of [11],[43],[44],[48]–[50])	To 18 mo postpartum: 83%

a Proportion of pregnant women accessing ANC, HIV testing for those in ANC, and receipt of HIV test result for those tested.

b Proportion of ANC sites with access to medications for PMTCT. This proportion is back-calculated in order to reach the reported POP for each scenario.

c Of women offered ARVs for PMTCT, the proportion remaining in care during the antenatal period, used as a proxy for acceptance of and adherence to medications. Retention in care postpartum: Of all postpartum women, the proportion linking to HIV care by the 6-wk postpartum visit. Impacts on MTCT of loss to follow-up after 6 wk postpartum, in the absence of specific data, are incorporated into highest-risk transmission estimates.

d Proportion of patients receiving care at all stages of the PMTCT cascade, defined as the product of (drug availability)×(care and testing)×(retention).

### Model Structure

#### Antenatal and intrapartum outcomes: MTCT model

The MTCT model is a previously published, decision-analytic simulation of a cohort of pregnant women from the time of conception through delivery (TreeAgePro) [28],[29]. The model uses a decision-tree (deterministic) structure, including probabilities of the following: presentation to ANC; offer and acceptance of HIV testing; receipt of HIV test results; clinical assessment for ART eligibility; CD4 testing and receipt of results; offer of, acceptance of, and adherence to ARVs for PMTCT; maternal mortality during pregnancy; HIV testing in labor for women with unknown or negative HIV status; live birth; infant HIV infection by the time of delivery; and linkage to postnatal care and ART for mothers and infants (Figure A in Text S1).

#### Postnatal outcomes: CEPAC adult model

The CEPAC model of adult HIV infection is a computer simulation model of HIV disease in adults. Technical details of the CEPAC model are described in Text S1 [30],[38]; outcomes of the CEPAC model for postpartum women have been validated against published data (Text S1) [32]. For this analysis, the adult CEPAC model was used only to project maternal mortality risks during the first 2 y after delivery, in order to inform infant mortality rates and duration of breastfeeding in the infant model.

#### Postnatal outcomes: CEPAC infant model

A first-order, Monte Carlo simulation model of infant HIV infection and survival was added to the adult CEPAC model (Figure B in Text S1) [29]. Infants enter this model at birth and are assigned one of three HIV categories (HIV-unexposed, HIV-exposed but uninfected, or HIV-infected), as well as one of three maternal disease categories (HIV-uninfected, HIV-infected and “ART eligible,” or HIV-infected and “non-ART-eligible”). Over the first 2 y of life, modeled infants face a monthly probability of four key clinical events: (1) incident maternal HIV infection during breastfeeding, if mother was previously uninfected, causing infants to transition from “unexposed” to “exposed, uninfected”; (2) maternal death, with risks derived from the adult CEPAC model as above, after which infants are no longer at risk for HIV infection but are at higher risk of death due to orphanhood [39]–[42]; (3) infant HIV infection through breastfeeding, if infant was previously uninfected; and (4) infant death from any cause. Risks of infant mortality are stratified by infant HIV exposure and infection status, by receipt of ART if infected, and by maternal vital status.

The MTCT and CEPAC models were linked to allow a combined analysis in which each woman–infant pair is simulated together from the time of first presentation at ANC through pregnancy and delivery (the MTCT model), and then each woman and infant are simulated separately through the first 2 y postpartum (the CEPAC models). Details of the linkages between the CEPAC and MTCT models have been published previously [29] and are further described in Figures A and B in Text S1.

#### Loss to follow-up

During ANC in the MTCT model, women may be lost to follow-up between first ANC presentation and delivery [11],[24],[43]–[47], between delivery and 6 wk postpartum [11],[43],[44],[48]–[50], and after linkage to postnatal HIV care [43],[44],[51]–[56]. In the absence of data regarding rates of adherence to the Option A or Option B regimens during breastfeeding, the impacts of medication interruptions or discontinuations were assumed to be included in sensitivity analyses examining “highest risk” postnatal MTCT estimates.

### Model Input Parameters

Baseline maternal characteristics reflected cohorts of pregnant women in Zimbabwe (Table 2). At first ANC visit, mean age was 24 y (SD: 5 y) [57], and the proportion with CD4≤350/µl was 36%, based on data from Zimbabwe [58]. In the base case, MTCT risks were derived as the average (mean or midpoint) of risks reported in PTMCT studies in Africa (Table 3; Text S1) [4]–[10],[12]–[15],[58]–[69]. To avoid underestimating early postpartum MTCT risks, we included only data from breastfed infants, necessarily excluding pivotal PMTCT studies among replacement-fed populations [8],[70]–. In the absence of reported postnatal transmission risks for infants older than 6 mo of age who continue to breastfeed with ongoing prophylaxis (Option A or B), we assumed a constant monthly risk based on data in younger children, as has been observed for postnatal transmission without prophylaxis [73]. Infant mortality rates (Table 4) were derived from Joint United Nations Programme on HIV/AIDS HIV-deleted mortality estimates (HIV-unexposed infants) [74], the ZVITAMBO study in Zimbabwe (HIV-exposed, uninfected infants) [75], and pooled analyses of several African cohorts (HIV-infected infants) [26],[76],[77].

**Table 2:** Model input parameters: maternal characteristics and uptake of PMTCT services.

Variable	Base-Case Value	Range for Sensitivity Analyses	Data Sources
**Baseline maternal cohort characteristics**			
Age	24 (5)	20–30	MOHCW [57]
HIV prevalence	16%		MOCHW [36]
HIV incidence	1%/y		MOHCW [36]
Mortality during pregnancy	0.7%	0%–2%	MOHCW [36]
**Among HIV-infected women at first ANC visit**			
Proportion ART-eligible^a^	36%	0%–100%	ZVITAMBO trial [58]
CD4 count: total cohort	451 (50)		ZVITAMBO trial [58]
CD4 count: ART-eligible	275 (50)		ZVITAMBO trial [58]
CD4 count: non-ART-eligible	550 (50)		ZVITAMBO trial [58]
CD4 count: incident infection in pregnancy	664 (50)		MACS [92]
**Uptake of PMTCT services and postnatal care (equal for all coverage scenarios)**			
Sensitivity of clinical assessment of ART eligibility^a^	36%	20%–100%	MTCT-Plus cohort [80]
Delivery in health care facility	69%	50%–100%	MOHCW [36]
Probability of linking to pediatric HIV diagnosis, care, and ART	36%	0%–100%	WHO/United Nations Children's Fund [23]
Duration of breastfeeding (months)	12	0–18	WHO, ZVITAMBO trial [3],[58]

Age given as mean (SD) in years; CD4 counts given as mean (SD) in number/microliter. Maternal disease progression: details of the CEPAC model and data inputs describing maternal HIV disease progression with and without ART are provided in Text S1.

a ART eligibility defined as CD4≤350/µl or WHO stage 3/4 disease. In scenarios in which CD4 assays were not available, we simulated clinical assessment of ART eligibility. The sensitivity of clinical assessment of ART eligibility was reported for a CD4 threshold of 200/µl (36%); model sensitivity analyses using subsequent reports based on a CD4 threshold of 350/µl (sensitivity: 20%) did not substantially change the results [80].

MACS, Multicenter AIDS Cohort Study; MOHCW, Zimbabwe Ministry of Health and Child Welfare.

**Table 3:** Model input parameters: mother-to-child transmission risks.

Maternal HIV Status	PMTCT Regimen Received				
	No ARVs	sdNVP	Antenatal ZDV^a^	Extended Infant NVP	Triple-Drug Regimen
**Intrauterine/intrapartum period (one-time risks)**					
ART-eligible at conception	0.273 [60],[93] (0.199–0.322) [64],[93],[94]	0.176 [58],[79] (0.082–0.264) [4],[14],[65],[95]	0.136 [62] (0.091–0.157) [7],[96]	n/a	0.033 [7] (0.011–0.041) [6],[9],[13]
Non-ART-eligible at conception	0.175 [60],[93] (0.127–0.206) [64],[93],[94]	0.073 [58],[79] (0.033–0.109) [4],[14],[65],[95]	0.036 [62] (0.024–0.041) [13],[67],[96]	n/a	0.01 [9] (0.004–0.028) [8],[9]
Incident infection during pregnancy	0.30 (assumption)	0.20 (assumption)	0.16 (assumption)	n/a	0.033 (assumed = eligible)
**Postnatal period (rate/100 person-years, among infants HIV-uninfected at 4–6 wk of age)**					
ART-eligible	9.13 (EBF) [58],[79]; 15.43 (MBF) [58],[79] (5.73–28.36) [61],[94]	n/a	n/a	n/a	4.00 [67] (0–6.42) [7]–[9],[13]
Non-ART-eligible	2.86 (EBF) [58],[79]; 4.82 (MBF) [58] (1.79–8.82) [61],[69]	n/a	n/a	2.65 [5],[10] (1.44–3.74) [5],[10]	2.23 [9],[11],[12],[15] (0–6.42) [9],[10]
Incident infection during breastfeeding	9.13 (EBF) [58],[79]; 15.43 (MBF) [58],[79] (5.73–28.36) [61],[94]	n/a	n/a	n/a	4.00 [67] (0–6.42) [7]–[9] (assumed = eligible)

Data given as base-case value [references] (range for sensitivity analysis) [references].

a Antenatal ZDV reflects the antenatal component of the Option A regimen for women who are not eligible for ART. Per WHO 2010 PMTCT guidelines, the intrapartum sdNVP and 7-d postnatal ZDV/lamivudine “tail” components of the Option A regimen may be omitted if a woman receives>4 wk of antenatal ZDV. The cited transmission risks reflect a range of antenatal ZDV treatment durations, as well as studies both including and excluding the sdNVP and ZDV/lamivudine components. The Pediatric AIDS Clinical Trials Group Protocol 076 study [96] was conducted in a replacement-fed population. However, this study demonstrated MTCT risks at the upper bound of the published range, reducing concern for underestimation of early postpartum MTCT risk, and thus was used in the “highest risk” scenario.

EBF, exclusive breastfeeding (in first 6 mo of life, followed by mixed breastfeeding); MBF, mixed breastfeeding; n/a, not applicable.

**Table 4:** Model input parameters: infant mortality.

Variable	Base-Case Value	Range for Sensitivity Analyses	Data Source
Probability of live birth	95.7%–98%	95%–99%	MOHCW [57]
Relative increase in infant mortality if maternal death occurs	2-fold increase	2- to 6-fold increase	[39]–[42]
HIV-unexposed children	1-y risk: 5.4%		[74]
	2-y cumulative risk: 5.9%		
HIV-exposed, uninfected children	1-y risk: 7.4%		[75]
	2-y cumulative risk: 9.2%		
HIV-infected children, no ART: intrauterine/intrapartum infection	1-y risk: 51.0%		[26]
	2-y cumulative risk: 65.0%		
HIV-infected children, no ART: postpartum infection	1-y risk: 24.0%		[26]
	2-y cumulative risk: 38.0%		
HIV-infected children, on ART	1-y risk: 9.5%		[77],[97]
	2-y cumulative risk: 12.0%		

MOHCW, Zimbabwe Ministry of Health and Child Welfare.

### Model Validation and Sensitivity Analyses

Validation of model-derived MTCT and mortality risks against published data, and sensitivity analyses on many clinical and programmatic parameters, have been reported previously [29].

For this analysis, in addition to the impact of PMTCT uptake and regimen examined in the base-case analyses, we conducted univariate sensitivity analyses on three key factors influencing transmission risk. First, to examine the impact of the range of published MTCT risks for each regimen, we defined “highest risk” and “lowest risk” scenarios. The “lowest risk” scenarios use the lowest published MTCT risks for each drug regimen, reflecting the best reported field effectiveness or trial efficacy, and likely representing the most adherent study populations; the “highest risk” scenarios use the highest published MTCT risks for each drug regimen. Second, because the proportion of women with CD4≤350/µl or WHO stage 3/4 disease at first ANC visit has been reported to range widely (23%–68% [78],[79]), we varied this proportion in sensitivity analyses from 0% to 100%. Third, we investigated the contribution of prolonged breastfeeding by projecting MTCT risk at 4–6 wk of age (primarily reflecting transmission that occurs in the absence of breastfeeding), as well as after 18 mo of breastfeeding (the median duration reported in a cohort from Zimbabwe [58]), with ARV prophylaxis continued throughout breastfeeding.

We identified the parameters that exerted the greatest impact on MTCT risks by comparing the range of MTCT risks projected when each parameter was varied through clinically plausible ranges; the largest range of projected MTCT risks reflected parameters with the greatest influence on model results. We then performed multivariate sensitivity analyses to determine combinations of these factors necessary to reach MTCT risks less than 5%.

To further examine the multiplicative impact of individual components of the PMTCT cascade, we varied uptake at each step in the care pathway (access to ANC, HIV testing in ANC, receipt of HIV test results, medication availability, adherence to medications, and linkage to postnatal HIV-related care), as well as the availability of CD4 counts and ART for women with CD4≤350/µl.

## Results

### MTCT Risk among Infants Born to Women HIV-Infected Before Pregnancy (Cohort 1)

The modeled 12-mo MTCT risk in the sdNVP-based 2008 Zimbabwean National PMTCT Program was 20.3% (Table 5, top). Modeled improvements in PMTCT uptake in the 2009 National PMTCT Program are projected to have reduced this risk to 18.0%. If sdNVP could be replaced by WHO-recommended regimens at the current 56% uptake level, MTCT risk would be projected to fall to 14.4% with Option A, or 13.4% with Option B. The potential impacts of further improvements in coverage are highlighted in the “WHO target” and “optimal uptake” scenarios. If Option A were implemented at the WHO target level of 80%, MTCT risk would be projected to fall to 10.5%; “optimal” 95% uptake would reduce this further to 7.7%. With Option B, these risks are projected at 9.1% (80% uptake) and 6.1% (95% uptake). Similar impacts of PMTCT regimens and uptake scenarios are seen for the secondary outcomes of infant infection risk at 4–6 wk of age, 2-y pediatric survival, and 2-y pediatric HIV-free survival (Tables E–G in Text S1).

**Table 5:** Results of a model of PMTCT services in Zimbabwe: cumulative 12-mo infant HIV infection risks.

Uptake Scenario	Base-Case Model Results for Each PMTCT Regimen		
	sdNVP	WHO 2010 Option A	WHO 2010 Option B
**Infants of women HIV-infected before pregnancy: MTCT risk (%)**			
Zimbabwe National PMTCT Program 2008 (36%)	20.3	17.9	17.2
Zimbabwe National PMTCT Program 2009 (56%)	18.0	14.4	13.4
WHO target (80%)	15.4	10.5	9.1
Optimal (95%)	13.4	7.7	6.1
**Infants of annual cohort of pregnant women in Zimbabwe: proportion (%) of cohort (number of annual infections; reduction from 2009 program)**			
Zimbabwe National PMTCT Program 2008 (36%)	3.5 (13,910)	3.2 (12,380)	3.0 (11,950)
Zimbabwe National PMTCT Program 2009 (56%)	3.2 (12,440; reference)	2.6 (10,200; 18%)	2.4 (9,560; 23%)
WHO target (80%)	2.8 (10,790; 13%)	2.0 (7,750; 38%)	1.8 (6,890; 45%)
Optimal (95%)	2.4 (9,590; 23%)	1.5 (5,970; 52%)	1.3 (4,950; 60%)

### Population-Level Results for All Women Becoming Pregnant Each Year in Zimbabwe (Cohort 2)

For an annual pregnancy cohort in Zimbabwe, incorporating HIV-negative women, chronically HIV-infected women, and women with incident HIV infection (Table 5, bottom), the overall modeled 12-mo risk of infant HIV infection under the 2008 National PMTCT Program was 3.5%, representing 13,910 HIV-infected infants. Improvements in uptake are modeled to have reduced this risk to 3.2% (12,440 infections) by 2009, averting 1,470 infections. Replacement of sdNVP by Option A or B at the current uptake level would be projected to reduce this risk to 2.4%–2.6% (9,560–10,200 infections), and improved levels of uptake would reduce this modeled risk further, to 1.3% (4,950 infections, a 60% reduction in infant infections compared to sdNVP at 56% uptake) with Option B at “optimal” 95% uptake.

### Univariate Sensitivity Analyses

**Figure 2:**
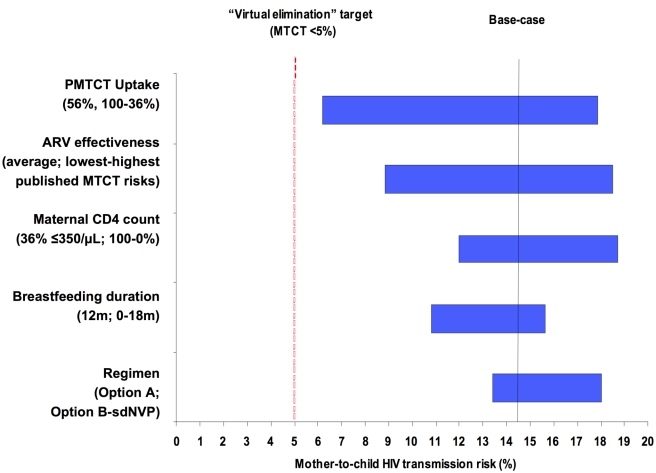
Key parameters determining MTCT risk. Tornado diagram summarizing the results of key one-way sensitivity analyses. Model parameters are on the vertical axis. For each parameter, the value used in the base-case analysis is listed in parentheses, followed by the range examined in sensitivity analysis. For example, the “regimen” provided for PMTCT is varied from Option B (lowest MTCT risk with all other parameters held constant), through Option A (base-case MTCT risk), to sdNVP (highest MTCT risk). The horizontal axis represents projected MTCT risk by the time of weaning. The solid vertical line represents transmission risk (14.4%) at the base-case set of parameters: 56% uptake, mean published MTCT risks, 36% of mothers with CD4<350/µl, breastfeeding duration of 12 mo, and the WHO “Option A” regimen. The dashed vertical line represents the 5% MTCT target of “virtual elimination” expressed by international HIV/AIDS agencies including WHO and the Joint United Nations Programme on HIV/AIDS. ARV prophylaxis in the Option A and Option B regimens is assumed to continue throughout the duration of breastfeeding.

Univariate sensitivity analyses investigating the individual impacts of PMTCT uptake, lowest/highest risk scenarios, maternal CD4 count, breastfeeding duration, and PMTCT regimen among Cohort 1 are shown in Figure 2. When individual parameters were varied from the base-case scenario (Option A regimen, 12 mo of breastfeeding, 36% of mothers ART-eligible, and average of published MTCT risks for each regimen), PMTCT uptake was the most influential single parameter (Figure 2, widest bar). Projected transmission, with all other parameters held equal, ranged from 6.2% (100% uptake), through 14.4% (56% uptake, indicated by “base-case projection line”), to 17.9% (36% uptake). The second most influential parameter was the range of published MTCT risks for each regimen (ranges shown in Table 3): with Option A at 56% uptake, 12-mo MTCT risks ranged from 8.8% (“lowest risk”) to 18.5% (“highest risk”). The individual impacts of PMTCT uptake and lowest/highest risk scenarios, as well as of maternal CD4 count and breastfeeding duration, were greater than the impact of the choice of PMTCT regimen (Figure 2, narrowest bar). Additional univariate sensitivity analyses suggested that variations in the availability of HIV testing in labor and sdNVP for women identified as HIV-infected during labor also did not change the policy conclusions (Text S1). Overall, these univariate sensitivity analyses demonstrated that optimizing any single parameter shown in Figure 2 did not reduce modeled MTCT risk to less than 5%, underscoring the need for improvements in multiple parameters simultaneously to approach the “virtual elimination” of HIV in Zimbabwe.

### Multivariate Sensitivity Analyses

**Figure 3:**
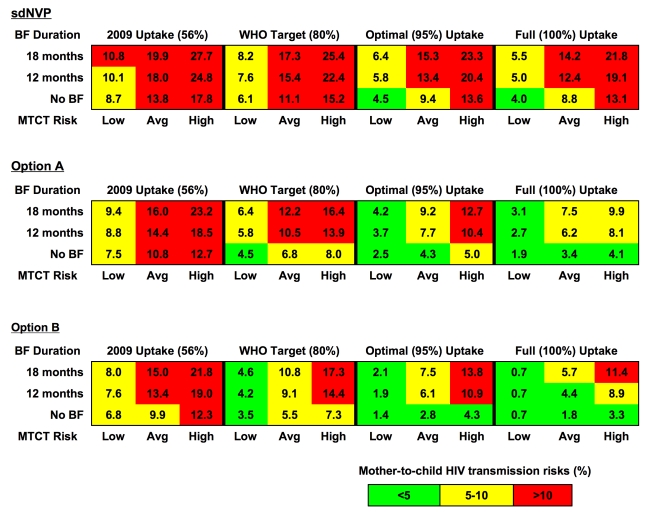
Combinations of parameters needed to achieve MTCT risks<5%, 5%–10%, and >10%. Each horizontal block represents results for a specific drug regimen: sdNVP (top), Option A (middle), and Option B (bottom). Within each block, four levels of uptake are depicted across the top horizontal axis: 56% uptake (current estimated uptake in Zimbabwe), 80% uptake (the WHO target), 95% uptake (reported in neighboring Botswana), and 100% uptake (to reflect maximum biologic efficacy of each regimen). The vertical axis illustrates three durations of breastfeeding (BF) for each modeled PMTCT regimen: 18 mo (median in Zimbabwe), 12 mo (concordant with 2010 WHO infant feeding guidelines), and no breastfeeding; ARV prophylaxis in the Option A and Option B regimens is assumed to continue throughout the duration of breastfeeding. The lower horizontal axis shows three categories of published MTCT risks for each drug regimen, including the lowest published risks, the average of published risks (the base-case parameters), and the highest published risks. The percentage in each cell reflects the MTCT risk associated with each set of parameters, and cells are color-coded to reflect broad categories of transmission. Red-colored cells indicate MTCT risks>10%, yellow-colored cells indicate MTCT risks between 5% and 10%, and green-colored cells indicate MTCT risks<5%.

Multivariate sensitivity analyses highlighted the combinations of factors necessary to reduce MTCT among Cohort 1 to the WHO target of less than 5% (Figure 3, regions shaded in green). For example, at 80% uptake of Option A (middle panel), only if the lowest published MTCT risks and avoidance of breastfeeding were assumed was the <5% target projected to be achieved. With 80% uptake of Option B, a projected risk less than 5% still required assumption of the lowest published MTCT risks, but permitted breastfeeding durations up to 18 mo. With both the lowest published transmission risks and “optimal” (95%) uptake, 12-mo MTCT risks could decrease to 3.7% (Option A) or 1.9% (Option B).

### Impact of Overall Product of Participation (Cohort 1)

When overall uptake by the time of delivery was held constant at any given level, the specific step in the cascade at which uptake was varied did not affect 4–6-wk MTCT risk (Table 6). In contrast, the proportion of women–infant pairs linking to postnatal care had a substantial impact on 12-mo MTCT risk, especially with Options A and B (Table 7).

**Table 6:** Impact of uptake at key antenatal steps in the PMTCT cascade on MTCT at 4–6 wk of age.

Proportion of Women Accessing Each Step in Cascade						MTCT at 4–6 wk
Access ANC	HIV Test	HIV Test Result	Drug Availability	Adherence	Total Uptake by Delivery	
91	87	99	82	87	56	13.8
56	100	100	100	100	56	13.8
100	56	100	100	100	56	13.8
100	100	56	100	100	56	13.8
100	100	100	56	100	56	13.8
100	100	100	100	56	56	13.8

Results are shown with 56% uptake, sdNVP strategy. All data given as percents.

**Table 7:** Impact of linkage to postnatal care by 6 wk postpartum, following 56% uptake at delivery.

Regimen	12-mo MTCT Risk (Percent) for Given Linkage to Postnatal Care					
	0% Linkage	25% Linkage	50% Linkage	75% Linkage	87% Linkage (Base Case)	100% Linkage
**sdNVP**	19.8	19.2	18.7	18.2	18.0	17.7
**Option A**	17.1	16.3	15.5	15.4	14.4	14.0
**Option B**	16.2	15.4	14.6	14.0	13.4	13.0

To isolate the impact of linkage on postnatal care, results are shown for the base-case scenario of antenatal PMTCT uptake (56% uptake at the time of delivery).

### Impact of Available CD4 Counts and ART for Women with CD4≤350/µl (Cohort 1)

In the base-case analysis, CD4 assays were assumed to be available only in the Option A and Option B strategies; for sdNVP, ART eligibility was assessed clinically, with a sensitivity of 36% for WHO stage 3/4 disease or CD4≤350/µl [80]. Furthermore, the proportion of women offered medications was assumed to be equal among identified ART-eligible women (offered ART) and all others (offered the sdNVP, Option A, or Option B regimens). Two modeled scenarios demonstrated the impact of improvements in CD4 and ART availability. Table 8 (top) depicts a scenario in which no CD4 assays were available, regardless of regimen. At 56% uptake of sdNVP or Option A, although 36% of the cohort was truly ART-eligible, only 7% of HIV-infected women were identified as ART-eligible and received ART. Table 8 (bottom) shows a scenario in which CD4 assays were available for all women identified as HIV-infected in ANC, and all women with CD4 values≤350/µl were offered ART. At 56% uptake of sdNVP or Option A, this scenario of CD4/ART availability led 25% of women to be identified as ART-eligible and to receive ART. Although the proportion of women receiving sdNVP or antenatal ZDV decreased from 49% to 31% (to maintain a total uptake of 56%), MTCT risks at 4–6 wk were substantially lower when CD4 assays were available. Notably, in the sdNVP strategy at 56% uptake, adding CD4 assays and ART for eligible women reduced 4–6-wk MTCT from 13.8% to 11.4%, a larger impact than that of replacing sdNVP with Option A without CD4/ART availability (4–6-wk MTCT risk of 12.0%). Under the Option B strategy, the effect of targeting ART to women with low CD4 counts was markedly smaller: at 56% uptake of Option B, prioritizing ART for women with CD4 values≤350/µl would reduce 4–6-wk MTCT from 9.9% to only 9.6%, by slightly shifting the distribution of women on three-drug ARVs from non-ART-eligible to ART-eligible women.

**Table 8:** Impact of availability of CD4 assays and ART for women with CD4≤350/µl.

Scenario	Uptake of Specific Regimens (Percent of Entire Cohort)				Outcomes	
	ART (Identified as ART-Eligible)	sdNVP	ZDV	Total Uptake, Any Regimen	MTCT at 4–6 wk	MTCT at 12 mo^a^
**Clinical assessment of ART eligibility (CD4 assay not available); equal availability of ART for women identified as ART-eligible and of ARVs for all others**						
**56% uptake**						
sdNVP	7	49	0	56	13.8	18.0
Option A	7	0	49	56	12.0^b^	15.6^b^
Option B	20	0	0	56	9.9	13.4
**80% uptake**						
sdNVP	10	70	0	80	11.1	15.0
Option A	10	0	70	80	8.6	11.6
Option B	29	0	0	80	5.5	8.5
**95% uptake**						
sdNVP	12	83	0	95	9.4	13.3
Option A	12	0	83	95	6.4	9.6
Option B	34	0	0	95	2.8	5.9
**CD4 assay available for all women identified as HIV-infected, and ART available for all women with observed CD4≤350/µl**						
**56% uptake**						
sdNVP	25	31	0	56	11.4^b^	15.8^b^
Option A	25	0	31	56	10.3	14.0
Option B	25	0	0	56	9.6	13.1
**80% uptake**						
sdNVP	36	44	0	80	7.6	11.7
Option A	36	0	44	80	6.1	9.3
Option B	36	0	0	80	5.0	8.1
**95% uptake**						
sdNVP	36	59	0	95	6.2	10.3
Option A	36	0	59	95	4.1	7.4
Option B	36	0	0	95	2.6	5.8

a Results highlight that providing CD4 assays for all women identified as HIV-infected, and ART for all women with CD4≤350/µl would lead to projected MTCT risks under the 2009 sdNVP-based program (56% uptake, sdNVP strategy: 11.4% at birth and 15.8% at 12 mo) comparable to if Option A were implemented at 56% uptake without increased CD4 and ART availability (56% uptake, Option A strategy: 12.0% at birth and 15.6% at 12 mo).

## Discussion

We present results from a validated computer simulation model of MTCT in Zimbabwe, a setting where high HIV prevalence, prolonged breastfeeding, and limited resources make the WHO 2010 PMTCT guidelines both critical and challenging to implement. These analyses enumerate the potential benefits of improved PMTCT services along two dimensions: implementation of the WHO-recommended Option A or B regimens, and improved uptake of any given regimen at each step in the PMTCT cascade (Figure 1). Our results are similar to those from prior analyses using different modeling methodologies, including the analyses from which the initial targets were generated; consistent findings suggest that “virtual elimination” will require massive scale-up of PMTCT services [22],[81]. Although the WHO target may therefore seem difficult to reach, the WHO's “3 by 5” campaign (to deliver ART to 3 million HIV-infected patients by 2005) demonstrated the role that such ambitious public health goals can play in catalyzing efforts to dramatically expand HIV/AIDS services [82]. In addition, the analyses presented in this article simultaneously examine PMTCT uptake, breastfeeding duration, the range of MTCT risks published for each regimen, and both Options A and B from the 2010 WHO PMTCT guidelines, to identify combinations of factors that will facilitate achieving MTCT risks of less than 5% and the “virtual elimination” of pediatric HIV.

Most importantly, we find that improvements in PMTCT uptake are critical to approaching the goal of virtual elimination of MTCT. For example, a marked increase in PMTCT uptake occurred in Zimbabwe between 2008 (36%) and 2009 (56%), despite a period of economic hyperinflation [83]; as a result, projected MTCT risk was reduced from 20.3% to 18.0%, and an estimated 1,470 additional new infections were averted in a single year. Furthermore, once sdNVP is replaced by either WHO-recommended regimen, the level of PMTCT uptake exerts a far greater impact on MTCT rates than does the specific choice between Option A and Option B. Building from the 2009 National PMTCT Program in Zimbabwe, replacing sdNVP with Option A at 56% uptake (MTCT risk, 14.4%) would be more effective than expanding sdNVP uptake to 80% (MTCT risk, 15.4%). However, once the program reaches 56% uptake of Option A, improving uptake of Option A to 80% (MTCT risk, 10.5%) is projected to result in better outcomes than would replacing Option A with Option B at current uptake (MTCT risk, 13.4%). Notably, even with 95%–100% uptake, there are very few scenarios in which MTCT risk is estimated to fall below the 5% “virtual elimination” target threshold set by the WHO (Figure 3). With 12 mo of breastfeeding and average published MTCT risks for each regimen, nearly 100% uptake of Option B would be required to reach MTCT risks of less than 5%.

The results of this analysis depend critically on the modeled effectiveness of each ARV regimen, leading to two key conclusions. First, Option B may not be superior to Option A in preventing MTCT. The ranges of published MTCT risks for Options A and B are wide and overlapping (Table 3), and no randomized comparison of Option A with Option B has yet been reported. While data from available trials are limited by differences in populations and interventions [4]–[15],[67],[84], they suggest that Options A and B might have similar efficacy among women with CD4>350/µl, supporting WHO's equally strong recommendation of both regimens [21]. Results of a multi-national clinical trial comparing these regimens in women with CD4>350/µl will critically inform policy decisions around Options A and B [85].

Second, the lowest published MTCT risks for each regimen likely reflect an additional contribution of medication adherence, beyond that explicitly included in our MTCT model. For example, the lowest reported MTCT risks with maternal triple-drug ARV regimens in breastfeeding suggest 0% transmission by 6 mo of age, even among women with CD4<200/µl [9], which is lower than other reported risks of 0.5%–2.9% [6],[7],[10]–[13],[15]. Zero transmission was observed in a trial in which 92%–99% of women achieved viral suppression (HIV RNA<400 copies/ml) at delivery and 6 mo postpartum, suggesting that sustained adherence might have been greater in this trial than in trials reporting higher MTCT rates without such suppression data [9]. Among mother–infant pairs attempting to complete the Option A or Option B regimens beyond 6 mo of breastfeeding, adherence has not yet been reported. Based on high reported loss to follow-up rates among HIV-infected adults on ART, particularly postpartum women, as well as anecdotal evidence of the challenges of administering daily medications to otherwise healthy infants, it might be anticipated that the adherence that is observed when Options A and B are implemented in treatment programs will be lower than the trial-based adherence rates that are reflected in our base-case and “lowest risk” scenarios [43],[86].

In addition to the range of published MTCT risks for each regimen, reduced duration of breastfeeding is a key parameter that might permit the risk of MTCT to approach 5%. In settings where replacement feeding is the norm, MTCT risks have been reduced to 0%–2% [1],[2]. However, in Zimbabwe, as in many other resource-limited settings, infant formula milk may not be available, affordable, socially acceptable, or safe (due to lack of clean water or facilities for clean formula preparation) [3]. Replacement feeding, while reducing HIV transmission risk, therefore confers high risks of diarrheal disease, malnutrition, and mortality [3],[8]. Interventions to safely reduce breastfeeding duration in such settings, such as improved societal infrastructure for safe water systems, would likely confer tremendous health benefits, extending far beyond the infants of HIV-infected women, but will be costly and challenging to implement [87]. In the interim, our results suggest that promotion of high levels of access to postnatal care and adherence to ARV prophylaxis during breastfeeding will be critical to reduce the risk of MTCT to less than 5%.

Although PMTCT uptake in low- and middle-income countries has risen from 10% in 2004 to 53% in 2009 [19],[37], greater access to care and medications for HIV-infected pregnant women is still needed. The PMTCT “cascade” of care comprises multiple steps at which pregnant and breastfeeding women and their infants can be lost from care, and overall uptake is reduced multiplicatively at each step [22],[24],[25],[88]. For example, considering eight steps (access to ANC, HIV testing, receipt of test result, CD4 testing, receipt of CD4 result, adherence/retention before delivery, presentation to postnatal care, and adherence to breastfeeding prophylaxis regimens), each with the 92% average uptake observed in the PEARL multi-country study [24], the proportion of women completing care may be as low as 0.92^6^ = 61% by delivery, or 0.92^8^ = 51% by weaning. Notably, we find that the specific step in the antenatal PMTCT cascade at which improvements are made does not impact the risk of MTCT, if uptake is improved equally among ART-eligible and non-ART-eligible women. However, large reductions in the risk of MTCT are projected to occur if PMTCT uptake is increased by improving the proportion of ART-eligible women who receive ART (for example, through greater availability of CD4 testing and timely CD4 result return, or through selection of the Option B strategy). In addition, interventions to improve linkage to postnatal maternal HIV-related care (and ART for ART-eligible breastfeeding mothers) may substantially reduce postnatal HIV transmission. A focused operational research agenda is critical to identify, implement, and evaluate interventions that improve uptake and retention at each step in the PMTCT cascade, with a particular focus on availability of CD4 assays and ART for women with low CD4 counts [89].

There are three main limitations to this analysis. First, computer models simplify complex biologic and operational processes. Second, data from multiple sources were necessarily combined. However, sensitivity analyses were conducted to examine the effects of variation in key simplifying assumptions, including CD4 assay availability and the availability of HIV testing and sdNVP during labor, as well as wide ranges for each model input parameter. We found that these assumptions and data inputs had no substantive impact on policy conclusions, except where highlighted in the Results and Discussion. Finally, we report only short-term pediatric outcomes, and exclude potential impacts on maternal or community health resulting from expansion of PMTCT programs. In addition to a reduction in infant HIV infections, many other benefits may result from the planned transition from sdNVP to Option A or B, including the avoidance of exposure to sdNVP and the associated risk of drug-resistant virus for both mothers and infants [90],[91], as well as long-term maternal and pediatric health benefits associated with the monitoring and treatment infrastructure required for implementation of more complex PMTCT regimens. The potential health benefits and costs of these “indirect effects” will comprise important areas of future research.

### Conclusions

In Zimbabwe, the planned implementation of the 2010 WHO PMTCT guidelines with Option A is projected to substantially reduce infant HIV infection risk compared to the 2009 national program. In order to approach the goal of “virtual elimination” of pediatric HIV (MTCT risk less than 5%), a national program based on either Option A or Option B will also need to include strategies to improve PMTCT uptake to nearly 100% throughout pregnancy and breastfeeding, to safely reduce the duration of breastfeeding, and to support medication adherence (thereby maximizing the effectiveness of each ARV regimen) during both pregnancy and breastfeeding.

## Supporting Information

Text S1 **Additional information on methods and model structure.**
(DOC)

## Abbreviations

ANC: antenatal care
ART: antiretroviral therapy
ARV: antiretroviral drug
CEPAC: Cost-Effectiveness of Preventing AIDS Complications
MTCT: mother-to-child HIV transmission
NVP: nevirapine
PMTCT: prevention of mother-to-child HIV transmission
POP: product of participation
SD: standard deviation
sdNVP: single-dose nevirapine
WHO: World Health Organization
ZDV: zidovudine
