# Supplementary material for: What Will It Take to Eliminate Pediatric HIV? Reaching WHO Target Rates of Mother-to-Child HIV Transmission in Zimbabwe: A Model-Based Analysis
Source: PLoS Med. 2012 Jan 10;9(1):e1001156. doi: 10.1371/journal.pmed.1001156 (PMC3254654; doi:10.1371/journal.pmed.1001156)
Supplement: Text S1 — Additional information on methods and model structure. (DOC) [file pmed.1001156.s001.doc]

**What will it take to eliminate pediatric HIV?**

**Reaching WHO target rates of mother-to-child HIV transmission in Zimbabwe**

**Supporting Information**

**(Text S1)**

Andrea L. Ciaranello, MD, MPH, *et al.*

**INTRODUCTION**

# This supplemental information is included to provide methodologic details to support the description of the methods in the manuscript text, as well as additional model output and results.

**METHODS**

**PMTCT regimens evaluated**

The 2010 World Health Organization guidelines for prevention of mother-to-child transmission (PMTCT) of HIV are summarized in Table A. Specific details of the antenatal, intrapartum, and postpartum components of each of the modeled PMTCT regimens are outlined in Table B.

**PMTCT uptake scenarios**

The “care and testing” domain referred to the proportion of all pregnant women accessing antenatal care (ANC), the proportion of those in ANC who received an HIV test, and the proportion of those tested who received their HIV test results. “Drug availability” referred to the proportion of ANC sites at which ARVs for PMTCT were available. “Retention” referred to the proportion of patients remaining in care and adhering to the prescribed PMTCT regimen during two time periods: 1) between ANC booking and delivery, and 2) between delivery and 18 months postpartum.

To inform the rates of uptake at each domain in the PMTCT cascade, the following data sources were used:

- **Zimbabwe 2008 and 2009 uptake scenarios:** Data were derived from WHO publications reporting rates of ANC and PMTCT services by country and from detailed local data collected by the Zimbabwe Ministry of Health . Where not available from Zimbabwe (retention in antenatal care and linkage to postnatal care, the mean of reported values for sub-Saharan Africa was used . For the 2009 Zimbabwe scenario, this mean value could not be combined with estimates in the care and testing domain (even with 100% drug availability) to reach an overall uptake of 56% at the time of delivery; an adherence estimate from a detailed patient tracing study in Uganda (87%) was used instead .
- **WHO target:** Estimates are based on target levels of PMTCT coverage stated by WHO and UNICEF in 2009 .
- **95% uptake scenario:** This scenario is not based on reported data for each domain of the cascade, but instead on an overall uptake of 95%, as has been reported for Botswana .
- **100% uptake scenario:** A scenario of 100% uptake of all stages of the PMTCT cascade was evaluated to project the lowest possible MTCT risks achievable with the evaluated PMTCT regimens.

To permit calibration to published uptake levels in the base-case analyses, two assumptions were made: 1) HIV testing in labor and sdNVP for women diagnosed with HIV infection in labor were assumed unavailable, and 2) all women identified as HIV-infected in the Option A regimen were assumed to undergo CD4 testing and receive CD4 results with sufficient time to select an antenatal ARV regimen. Both assumptions were investigated in sensitivity analyses.

**Model structure and validation**

MTCT model

Details of the decision-tree MTCT model structure and validation of the model against published data have been reported previously, and are also summarized here . Modeled steps in the PMTCT cascade, depicted in Figure A, include: presentation to ANC; offer and acceptance of HIV testing; receipt of HIV test results; clinical assessment for ART eligibility; CD4 testing and receipt of results; offer of, acceptance of, and adherence to ARVs for PMTCT; maternal survival of pregnancy and childbirth; delivery of a live infant; HIV infection status in the infant at birth; linkage to postnatal care and ART for mothers; and linkage to HIV care and ART for infants. Probabilities of HIV transmission by 4-6 weeks of age (reflecting in utero and intrapartum HIV transmission) are stratified by maternal HIV stage (“ART eligible,” defined as above, or “non-ART-eligible”), and by ARV regimen received for PMTCT.

In the model, if loss to follow-up occurs during the antenatal period, no antenatal ARVs are received, but the opportunity to access HIV testing and sdNVP in labor remains (for this analysis, these opportunities were included in sensitivity analyses only). Incident infections in late pregnancy may be detected via HIV testing in labor, if available; incident infections during breastfeeding are not detected, nor breastfeeding prophylaxis initiated, unless women present to care with severe opportunistic infections (at which time ART is initiated).

CEPAC adult model

For this analysis, the adult CEPAC model was used only to determine the risk of maternal mortality during the first two years after delivery. Five discrete cohorts of postpartum women were simulated: ART-eligible and in HIV care (therefore receiving ART), non-ART-eligible and in care (thus receiving either extended NVP prophylaxis for infants (Option A) or breastfeeding-limited triple-drug ARV regimens (Option B), and initiating maternal ART when becoming ART eligible), ART-eligible and not in care (returning to care with development of a severe opportunistic infection), non-ART-eligible and not in care, and incidently infected during breastfeeding.

The adult CEPAC model is a first-order, stochastic, Monte Carlo simulation of HIV infection. The structure and technical details of the model have been previously published and validated against clinical data for postpartum women, as well as non-postpartum men and women . Details of the model are summarized here; for additional information, we refer the reader to the detailed Technical Appendices of prior publications , as well as to the CEPAC website (http://web2.research.partners.org/cepac).

*Model overview.* HIV-infected women enter the CEPAC model immediately after delivery and are simulated individually from model entry through death. Disease progression is characterized by a sequence of monthly transitions between health states; these include acute opportunistic and other infections prevalent in Southern Africa, chronic HIV infection, and death. The model records all clinical events and costs during each patient’s lifetime. Following each model simulation, summary statistics are calculated for the cohort of simulated women, including 2-year mortality risk (used for this analysis), as well as risks of opportunistic infections, life expectancy, and average lifetime healthcare costs. A cohort of ten million women is simulated to produce stable estimates of outcomes.

*Maternal cohort characteristics and disease progression without ART.* At model entry, women are assigned a baseline HIV RNA level (drawn from the distribution observed in the Cape Town AIDS Cohort, Table C, Section I ) and a baseline CD4 cell count (drawn from the distribution observed in the ZVITAMBO trial, Manuscript Table 1 ). In the absence of effective ART, current HIV RNA level determines the modeled rate of CD4 count decline (Table C, Section II). Current CD4 count, opportunistic infection (OI) prophylaxis, and history or absence of previous OIs determine the monthly risk of OIs and HIV-related death. HIV-related risks of death in the CEPAC model include mortality risks associated with acute opportunistic infection and chronic HIV-infection. Additional risks of death are derived from age- and gender-specific Zimbabwean mortality rates .

*Opportunistic infection prophylaxis.* In addition to antiretroviral therapy, all simulated patients receive trimethoprim-sulfamethoxazole as prophylaxis against *Pneumocystis jiroveci* pneumonia and other bacterial infections, and continue this therapy lifelong . The impact of trimethoprim-sulfamethoxazole prophylaxis on risks of clinical events, including medication toxicities, are described in Table C, Section III).

*Antiretroviral therapy.* With effective ART, modeled HIV RNA suppression leads to CD4 count increases, reducing the monthly risks for OIs and death. Virologic failure on ART may occur either “early” (≤24 weeks) or “late” (>24 weeks) after ART initiation. For patients with early or late virologic failure, CD4 counts decline, accompanied by increased risks of OIs and death. Patients who remain on ART despite virologic failure experience lower risks of OIs and death than do patients who discontinue ART, reflecting the CD4-independent benefit of ART .

In this analysis, based on 2009 Zimbabwean guidelines, women in HIV-related care were assumed to undergo outpatient clinical evaluation every two months and CD4 monitoring biannually; HIV RNA monitoring was assumed to be not available . ART regimens reflected 2009 Zimbabwean guidelines and common current practice in Zimbabwe (Table C, Section IV). If initiated during pregnancy, the first-line ART regimen included nevirapine with lamivudine and zidovudine; if initiated postpartum, first-line ART included nevirapine, lamivudine, and stavudine . The second ART regimen, if needed, included lopinavir/ritonavir with tenofovir/emtricitabine . Following WHO recommendations, simulated patients were switched from the first to second antiretroviral regimen after observed clinical or immunologic failure, defined as at least one severe OI, a ≥50% decrease from the peak on-ART CD4 count, or an absolute CD4 count <100 cells/µL . Opportunistic infections diagnosed during the first six months of therapy are not considered as criteria for switching or discontinuing therapy, to allow adequate time for the immunologic benefit of ART to develop.

CEPAC infant model

The structure of the CEPAC infant model is described in the Methods section of the primary manuscript. In addition, technical details of the model structure, model validation, and linkages to the MTCT model, as well as extensive univariate and multivariate sensitivity analyses, have been published previously .

Linkages between the MTCT, CEPAC adult, and CEPAC infant models

The MTCT model was linked to the CEPAC adult and CEPAC infant models, in order to simulate each woman-infant pair together through pregnancy and delivery, then separately through the first two years postpartum. This linkage was accomplished by first simulating maternal and infant outcomes in the appropriate CEPAC models, then using CEPAC model results (postnatal infection risk, survival, and HIV-free survival) as inputs to the MTCT model (Figure A).

**Model input parameters**

Key model input parameters are presented in Manuscript Table 1 and Table C.

*Maternal cohort characteristics and HIV disease progression.* Mortality risks for HIV-uninfected women, as well as HIV-unrelated mortality risks among HIV-uninfected women, were derived from UNAIDS cause-deleted mortality rates . For HIV-infected women, because detailed clinical data to inform HIV disease progression with and without ART were not available from Zimbabwe, risks of HIV-related morbidity and mortality were derived from a clinical cohort in South Africa (Table C) . Mean CD4 cell count for chronically and acutely infected mothers are shown in Manuscript Table 1, and modeled rates of maternal HIV disease progression, ART response, and mortality are described in Table C.

*Mother-to-child transmission risks.* MTCT risks during the intrauterine and intrapartum period (before 4-6 weeks of age) and postpartum period (4-6 weeks-18 months) were stratified by maternal disease status and by PMTCT regimen received (Manuscript Table 1). Postpartum transmission risks were additionally stratified by whether breastfeeding during the first six months of life was exclusive (EBF, 7% of cohort) or mixed (MBF, including any non-breastmilk liquid or solid, 93% of cohort) . In the base case, “average” MTCT risks were determined as follows: if at least five studies were relevant to a given parameter, the base-case estimate was calculated as the mean of all values except the highest and lowest values. If fewer than five studies contributed data, the base-case estimates was the study value closest to the midpoint of the published range, excluding studies reporting the highest and lowest values. The “lowest” and “highest” risks were derived from the lower and upper bounds of the published ranges for each PMTCT regimen, respectively.

*Pediatric survival and access to care*. Mortality estimates for HIV-unexposed children were derived from UNAIDS HIV-deleted mortality estimates , and mortality rates for exposed-uninfected infants from the ZVITAMBO study in Zimbabwe (Manuscript Table 1, Section IId) . Mortality rates for HIV-infected children, stratified by timing of HIV infection and/or ART treatment status, were derived from pooled analyses of multiple African cohorts . For infants of any HIV infection or exposure status, maternal death was assumed to increase subsequent mortality risks by two-fold . The probability of linking to HIV care and ART for infected infants was 36%, based on the 2009 WHO estimate of pediatric ART availability in Zimbabwe (Manuscript Table 1, Section IIb) .

**RESULTS**

**Base case results (Tables D-F)**

Base-case projections of MTCT risk after 12 months of breastfeeding are shown in the main manuscript, with additional detail regarding the impact of maternal CD4 count on this outcome in Table D. We also examined risk of MTCT at 4-6 weeks of age, reflecting primarily intrauterine and intrapartum HIV transmission (Table E), as well as 2-year pediatric survival (Table F) and

2-year pediatric HIV-free survival (Table G).

**Sensitivity analyses**

Key sensitivity analyses are described in the main manuscript. These include the impact of breastfeeding duration, maternal CD4 cell count, and range of published efficacies for each PMTCT regimen. In addition, we examined in sensitivity analyses the impact of full (100%) uptake of each regimen at each step in the PMTCT cascade (Tables D-G).

To better reflect common PMTCT practices in many settings, we also investigated scenarios in which: 1) all women with negative or unknown HIV status underwent HIV testing in labor, 2) all women identified as HIV-infected during labor received sdNVP, and 3) in the Option A regimen, reduced rates of CD4 testing (0-100%) and CD4 result return (0-100%) were simulated . Results are shown in Table H.

**Figure A. PMTCT "cascade;" schematic representation of the MTCT model (adapted with permission from Ciaranello *et al, PLoS ONE*, 2011; 6(6)).**

**LEGEND:** The MTCT model is a decision tree, coded in TreeAgePro software. Pregnant women enter the model at conception. The five modeled PMTCT strategies are shown at the decision node, indicated by a square. Circles indicate chance nodes, at which events occur based on probabilities derived from published literature. Triangles indicate terminal nodes, representing the clinical outcome of any single pathway through the model. Brackets reflect that the subsequent events emerging to the right of the bracket may follow any of the prior chance nodes included to the left of the bracket. At each chance node, the probabilities of all subsequent modeled events may depend on the PMTCT strategy being simulated and on the prior events leading to that node.

For each modeled PMTCT strategy, the series of events shown in the Figure may occur. For example, women may be HIV-infected or HIV-uninfected at conception (this probability is the HIV prevalence in antenatal care). If HIV-infected, they may be ART-eligible (CD4≤350/µL or WHO Stage 3-4 disease) or non-ART-eligible; ART-eligibility may be identified by CD4 testing, identified by clinical evaluation, or not identified. All women may access ANC, undergo HIV testing in ANC, and receive HIV test results, or may fail to access these steps in the cascade.

If identified as HIV-infected (correctly or incorrectly), women may be offered ARVs for PMTCT according to the PMTCT strategy being simulated, as well as ART if identified as ART-eligible (not shown). Probabilities for surviving pregnancy depend on receipt of ART; if maternal death occurs, infant death also occurs. Women who survive pregnancy may deliver at a healthcare facility or at home; if they deliver in a healthcare facility, they may access HIV testing (if previous status was unknown or negative), and if identified as HIV-infected at that time, may receive sdNVP in labor. All women surviving pregnancy then experience probabilities of live birth and HIV infection in the infant, depending on PMTCT regimen received. Finally, women may link or fail to link to postnatal HIV-related care for themselves.

At the end (far right) of any given path through the model, there are two sets of outcomes: infant outcomes and maternal outcomes. Only infant outcomes are considered in this analysis; they include HIV infection status (infected or uninfected at birth, shown), risk of postnatal HIV infection if uninfected at birth, and 2-year survival. These outcomes are derived from the CEPAC infant model, through specific simulations in the CEPAC infant model of each possible scenario described at the end of the pathways shown in the MTCT model. As an example of infant outcomes from the CEPAC infant model, an HIV-uninfected infant with an ART-eligible mother who is in postnatal care (and thus on ART) would face monthly risks of HIV infection based on receipt of maternal ART during breastfeeding (details shown in Figure B2, below). These CEPAC model outputs are then used as “payoffs” (outcomes) in the MTCT model, according to conventional methods for evaluation of a decision tree. The average value assigned to any modeled PMTCT strategy in the MTCT model is, in essence, a weighted average of the value of these outcomes at the end of each pathway (weighted by the probabilities of reaching each possible path endpoint).

**Figure B. Schematic representations of the CEPAC adult and infant models (adapted with permission from Ciaranello *et al, PLoS ONE*, 2011; 6(6)).**

Figure B1: Adult CEPAC model

Figure B2: Adult CEPAC model

**LEGEND**. Figure B shows schematic representations of the adult and infant CEPAC model structures.

Women enter the adult model (Figure B1) after delivery, either during acute infection (incident infection during the last trimester of pregnancy) or chronic HIV infection. They then face monthly risks of clinic events including opportunistic infections, medication toxicities, and death; these risks are stratified by the parameters listed in the Figure. For this analysis, the adult CEPAC model was used only to generate mortality rates for women in the first two years after delivery (these, in turn, influence HIV infection and mortality risks for infants in the infant CEPAC model, as described below).

Infants enter the infant model (Figure B2) after birth, either as HIV-unexposed infants (mothers are uninfected), HIV-exposed but uninfected infants, or infants infected during the intrauterine/intrapartum period. Unexposed infants become exposed if maternal incident HIV infection occurs during breastfeeding. Exposed-uninfected infants face a monthly risk of breastfeeding transmission; this risk ceases at weaning or if maternal death occurs. From any infection state, infants face a risk of all-cause mortality. Monthly risks of infant HIV infection and infant mortality are stratified by the parameters listed in the Figure.

**Table A**. Revised WHO guidelines for prevention of mother-to-child transmission (PMTCT) and regimens evaluated in a simulation model of PMTCT in Zimbabwe

| **A. 2010 World Health Organization PMTCT guidelines (summary)** |
| --- |
| 1. All women should undergo HIV testing during pregnancy |
| 2. Because clinical assessment of HIV disease stage is insensitive, women identified as HIV-infected should have CD4 cell counts measured to determine degree of immunosuppression. |
| 3. For women with CD4 ≤350/µL, combination (3-drug) ART should be initiated both for treatment of maternal HIV disease and for PMTCT, and should be continued lifelong after pregnancy and breastfeeding. |
| 4. For women with CD4 >350/µL, country-level decisions should be made between two options:   - “Option A:” zidovudine-based regimen from 14 weeks of gestation (or first presentation to antenatal care if after 14 weeks); for breastfed infants, extended daily nevirapine prophylaxis throughout breastfeeding. - “Option B:” maternal triple ARV prophylaxis from 14 weeks of gestation (or first presentation to antenatal care if after 14 weeks) through pregnancy and breastfeeding, to be discontinued after weaning. |

**Table B. PMTCT regimens simulated in a computer model** of PMTCT services in Zimbabwe

| **PMTCT**  **Regimen** | **Antenatal**  **(maternal)** | **Intrapartum**  **(maternal)** | **Postpartum**  **(maternal)** | **Neonatal**  **(infant)** |
| --- | --- | --- | --- | --- |
| **sdNVP** | Stage 3-4 disease: ZDV/3TC/NVP from ≥28 weeks  Others: None | Stage 3-4 disease: ZDV/3TC/NVP from ≥28 weeks  Others: sdNVP | Stage 3-4 disease: ZDV/3TC/NVP from ≥28 weeks  Others: None | sdNVP |
| **WHO “Option A”** | *CD4≤350/µL:* ZDV/3TC/NVP from ≥14 weeks or first ANC visit | *CD4≤350/µL:* ZDV/3TC/NVP | *CD4≤350/µL:* ZDV/3TC/NVP through breastfeeding | *CD4≤350/µL*:  NVP x 6 weeks |
|  | *CD4>350/µL:*  ZDV from ≥14 weeks or first ANC visit | *CD4>350/µL:* sdNVP  + ZDV/3TC * | *CD4>350/µL:* ZDV/3TC x 7days* | *CD4>350/µL:*  NVP through BF |
| **WHO “Option B”** | Regardless of CD4: ZDV/3TC/NVP from ≥14 weeks or first ANC visit | Regardless of CD4: ZDV/3TC/NVP | Regardless of CD4: ZDV/3TC/NVP through breastfeeding | Regardless of CD4:  NVP x 6 weeks |

**ARVs:** antiretroviral drugs; **sdNVP**: single-dose nevirapine; NPV: nevirapine; **ZDV**: zidovudine; **3TC**: lamivudine, **BF**: breastfeeding

The 2010 WHO PMTCT guidelines do not specifically address postnatal regimens for women who present late to antenatal care, and therefore receive only sdNVP as the antenatal/intrapartum component of their PMTCT regimens. In the “sdNVP” regimen, we assumed that women would not receive any postpartum medications (such as a ZDV/3TC tail or any extended postnatal prophylaxis), to reflect the 2002-2009 national PMTCT program. In the WHO 2010 guidelines regimens (Options A and B), we assumed that such women would link to postnatal HIV care at a rate based on published data. Women in postnatal HIV care would then undergo CD4 testing, and would initiate ART if CD4 ≤350/µL or WHO stage 3-4 disease was evident. Women found to have CD4 counts >350/µL would receive ART for breastfeeding prophylaxis (Option B) or their infants would receive extended NVP prophylaxis (Option A). For women with incident HIV infection during pregnancy or breastfeeding who link to postnatal care, we assumed that accurate diagnosis of infant infection status was made, such that under Options A and B, no breastfeeding prophylaxis was offered to mother-infant pairs in whom infants were infected before 4-6 weeks of age.

* Per 2010 WHO guidelines, sdNVP and intra/postpartum ZDV/3TC can be omitted if the mother receives >4 weeks of ZDV before delivery .

**Table C. Additional model input parameters: maternal HIV disease progression and response to therapy**

| **Variable** | **Value** |  | | **Data sources** |
| --- | --- | --- | --- | --- |
| **I. Baseline maternal cohort characteristics** | |  | |  |
| Distribution of initial HIV RNA (% total) | |  | | Cape Town AIDS Cohort |
| >100,000 copies/ml | 43 |  | |  |
| 30,001-100,000 copies/ml | 28 |  | |  |
| 10,001-30,000 copies/ml | 18 |  | |  |
| 3,001-10,000 copies/ml | 8 |  | |  |
| 501-3,000 copies/ml | 2 |  | |  |
| ≤ 500 copies/ml | 1 |  | |  |
| **II. Natural history of maternal HIV disease** | |  | |  |
| Mean monthly decrease in CD4/µL by HIV RNA | |  | | Multicenter AIDS Cohort Study |
| >30,000 copies/ml | 6.4 |  | |  |
| 10,001-30,000 copies/ml | 5.4 |  | |  |
| 3,001-10,000 copies/ml | 4.6 |  | |  |
| 501-3,000 copies/ml | 3.7 |  | |  |
| 0-500 copies/ml | 3.0 |  | |  |
| Monthly risk of severe opportunistic infections (%, range by CD4 count) | | | Cape Town AIDS Cohort | |
| WHO stage III-IV |  |  | |  |
| Visceral | 0.00-1.52 |  | |  |
| Non-visceral | 0.02-2.26 |  | |  |
| Non-specific | 0.00-0.71 |  | |  |
| Bacterial infection | 0.03-0.71 |  | |  |
| Tuberculosis | 0.16-1.96 |  | |  |
| Other severe infection | 0.14-1.67 |  | |  |

**Table C**, continued

| **Variable** | **Value** | |  | **Data sources** |
| --- | --- | --- | --- | --- |
| **II. Natural history of maternal HIV disease, continued** | | |  |  |
| Monthly risk of other clinical conditions (%, range by CD4 count) | | |  | Cape Town AIDS Cohort |
| Mild fungal infection | 1.76-3.14 | |
| Other mild infection | 2.33-2.67 | |
| Monthly risk of death from severe opportunistic infections (%) | | | | Cape Town AIDS Cohort |
| WHO stage III-IV |  | |  |
| Visceral | 9.21 | |  |
| Non-visceral | 2.38 | |  |
| Non-specific | 20.00 | |  |
| Bacterial infection | 2.94 | |  |
| Tuberculosis | 1.82 | |  |
| Other severe infection | 6.67 | |  |
| Monthly risk of HIV-related death (%, range by CD4 count) | | |  | Cape Town AIDS Cohort |
| No history of opportunistic infection | | 0.00-4.02 |  |
| With history of opportunistic infection | | 0.00-9.53 |  |
| Monthly risk of death from other clinical conditions (%) | | |  | Cape Town AIDS Cohort |
| Mild fungal infection | 0.54 | |  |
| Other mild infection | 0.39 | |  |
| Relative risk reduction on any ART regimen (%, range by CD4) | | | | Cotrimo-CI, ANRS 1203 |
| HIV-related death | 55-96 | |  |
| Acute opportunistic infections | 0-32 | |  |

**Table C**, continued.

| **Variable** | **Value** | |  | **Data sources** |
| --- | --- | --- | --- | --- |
| **III. Maternal trimethoprim-sulfamethoxazole effects** | | |  |  |
| % Reduction in probability of infection |  | |  | Cotrimo-CI |
| Mild bacterial diseases | 48.79 | |  |  |
| Invasive bacterial diseases | 49.81 | |  |  |
| WHO stage III-IV visceral diseases and other severe events | 17.88 | |  |  |
| Toxicity risk (%, one-time risk) |  | |  | Cotrimo-CI |
| Minor toxicity | 18.24 | |  |  |
| Major toxicity | 6.72 | |  |  |
| Maternal antiretroviral therapy |  | |  |  |
| **IV. Maternal ART effects** | | |  |  |
| Efficacy (% HIV RNA suppression at 24 weeks) ; gain in CD4/µL at 24 weeks on suppressive ART; yearly risk (%) of virologic failure >24 weeks after initiation) | | | | |
| 1^st^-line NVP/ZDV/3TC initiated in pregnancy | | 90%; 148;17.45% |  |  |
| 1st-line NVP/d4T/3TC post-partum | |  |  |  |
| With sdNVP exposure | | 85%; 148;17.45% |  | OCTANE trial ; |
| Without sdNVP exposure or if sdNVP followed short-course ZDV | | 90%; 148;17.45% |  |  |
| 2nd-line LPV/r/TDF/FTC (after failure of 1^st^-line regimen) | | 72%; 148;17.45% |  |  |
| **V. Loss to follow-up** | |  |  |  |
| Loss to follow-up from postnatal maternal care | | 16% (year 1);  6%/year (years 2+) | | Year 1:  Years 2+: |

**Table C, continued (footnotes):**

In the current analysis, the outputs of the CEPAC adult model (clinical outcomes for mothers after delivery) impact only risk of infant mortality.

**WHO**: World Health Organization; **NVP**: nevirapine; **d4T**: stavudine; **3TC**: lamivudine; **ZDV**: zidovudine; **LPV/r:** lopinavir/ritonavir; **TDF**: tenofovir; **FTC**: emtricibine; **scZDV**: short-course zidovudine.

**Table D**. Additional results of a model of PMTCT services in Zimbabwe: Infant infection risks at 12 months of age.

| **Uptake scenario** | **Base case model results for each PMTCT regimen (%)**  **(range, best- to worst-case MTCT risks)** | | | |
| --- | --- | --- | --- | --- |
|  | **sdNVP** | **WHO 2010 Option A** | **WHO 2010 Option B** | |
| **I. Infants of all women HIV-infected before pregnancy (36% ART-eligible): MTCT risk** | | | | |
| **Full uptake (100%) ^a^** | 12.4  (5.0-19.1) | 6.2  (2.7-8.1) | 4.4  (0.7-8.9) | |
| **II. Infants of non-ART eligible women, HIV-infected before pregnancy (CD4 > 350/µL, no WHO stage 3-4 disease): MTCT risk** | | | | |
| **Zimbabwe 2008 (36%)** | 16.8  (10.4.0-22.7) | 14.7  (10.0-18.4) | 13.6  ( 8.5-19.2) | |
| **Zimbabwe 2009 (56%)** | 15.0  (8.6-21.0) | 12.0  (8.0-15.1) | 10.4  (6.1-15.9) | |
| **WHO target (80%)** | 12.7  (6.5-19.0) | 8.9  (5.7-11.4) | 6.7  (3.2-12.1) | |
| **Optimal (95%) uptake** | 11.4  (5.2-17.7) | 6.7  (4.2-8.7) | 4.2  (1.3-9.5) | |
| **Full uptake (100%)** | 10.9  (4.7-17.3) | 5.8  (3.6-7.3) | 3.1  (0.4-8.4) | |
| **III. Infants of ART-eligible women, HIV-infected before pregnancy (CD4 ≤ 350/µL or WHO stage 3-4 disease): MTCT risk ^b^** | | | | |
| **Zimbabwe 2008 (36%)** | 26.5  (15.7-34.6) | 23.5  (14.2- 30.2) | 23.5  (14.2-30.2) | |
| **Zimbabwe 2009 (56%)** | 23.3  (12.7-31.4) | 18.7  (10.3-24.5) | 18.7  (10.3-24.5) | |
| **WHO target (80%)** | 20.0  (9.4-28.3) | 13.4  (6.0-18.4) | 13.4  (6.0-18.4) | |
| **Optimal (95%) uptake** | 17.2  (6.9-25.2) | 9.4  (2.9-13.4) | 9.4  (2.9-13.4) | |
| **Full uptake (100%)** | 15.2  (5.4-22.4) | 6.8  (1.2-9.7) | 6.8  (1.2-9.7) | |
| **IV. Infants of all pregnant women in Zimbabwe (HIV prevalence 16%, incidence 1%/year): proportion of annual cohort** | | | | |
| **Full uptake (100%)*** | 2.3  (1.0-3.4) | 1.3  (0.6-1.7) | | 1.0  (0.3-1.8) |

**Table D, continued (footnotes):**

a. 100% uptake results are provided for Sections I and IV, in addition to the four scenarios presented in the manuscript, to demonstrate the maximal projected impact of each regimen if medications could be administered to all HIV-infected women during pregnancy and breastfeeding.

b. Modeled outcomes of Option A and Option B are equivalent for women who are ART-eligible. This is because, in the base case, equal proportions of women who receive their HIV test result are identified as ART-eligible and initiated on ART (under Option A, because they receive a CD4 count and its result, and under Option B, because they receive ART as their PMTCT regimen).

**Table E. Results of a model of PMTCT services in Zimbabwe: Infant HIV-infection risks at 4-6 weeks of age**

| **Uptake scenario** | **Base case model results for each PMTCT regimen (%)**  **(range, best- to worst-case MTCT risks)** | | |
| --- | --- | --- | --- |
|  | **sdNVP** | **WHO 2010 Option A** | **WHO 2010 Option B** |
| **I. Infants of all women HIV-infected before pregnancy (36% ART-eligible): MTCT risk** | | | |
| **Zimbabwe 2008 (36%)** | 16.0  (10.8-19.9) | 14.1  (10.1-16.7) | 13.5  (9.6-16.4) |
| **Zimbabwe 2009 (56%)** | 13.8  (8.7-17.8) | 10.8  (7.5-12.7) | 9.9  (6.8-12.3) |
| **WHO target (80%)** | 11.1  (6.1-15.2) | 6.8  (4.5-8.0) | 5.5  (3.5-7.3) |
| **Optimal (95%) uptake** | 9.4  (4.5-13.6) | 4.3  (2.5-5.0) | 2.8  (1.4-4.3) |
| **Full uptake (100%)** | 8.8  (4.0-13.1) | 3.4  (1.9-4.1) | 1.8  (0.7-3.3) |
| **II. Infants of non-ART eligible women, HIV-infected before pregnancy (CD4 > 350/µL, no WHO stage 3-4 disease): MTCT risk** | | | |
| **Zimbabwe 2008 (36%)** | 13.2  (9.0-16.4) | 12.0  (8.7-14.1) | 11.1  (8.0-13.7) |
| **Zimbabwe 2009 (56%)** | 11.3  (7.2-14.6) | 9.3  (6.7-11.0) | 7.9  (5.6-10.3) |
| **WHO target (80%)** | 9.0  (5.0-12.4) | 6.1  (4.3-7.2) | 4.2  (2.8-6.2) |
| **Optimal (95%) uptake** | 7.5  (3.6-11.0) | 4.2  (2.8-4.8) | 1.8  (1.0-3.6) |
| **Full uptake (100%)** | 7.0  (3.2-10.5) | 3.5  (2.3-4.0) | 1.0  (0.4-2.8) |

**Table E, continued**

| **Uptake scenario** | **Base case model results for each PMTCT regimen (%)**  **(range, best- to worst-case MTCT risks)** | | |
| --- | --- | --- | --- |
|  | **sdNVP** | **WHO 2010 Option A** | **WHO 2010 Option B** |
| **III. Infants of ART-eligible women, HIV-infected before pregnancy (CD4 ≤ 350/µL or WHO stage 3-4 disease): MTCT risk** | | | |
| **Zimbabwe 2008 (36%)** | 21.0  (14.1-26.1) | 17.9  (12.6-21.2) | 17.9  (12.6-21.2) |
| **Zimbabwe 2009 (56%)** | 18.2  (11.4-23.4) | 13.3  (9.0-15.8) | 13.3  (9.0-15.8) |
| **WHO target (80%)** | 14.8  (8.1-20.3) | 7.9  (4.7-9.4) | 7.9  (4.7-9.4) |
| **Optimal (95%) uptake** | 12.7  (6.1-18.3) | 4.4  (2.0-5.4) | 4.4  (2.0-5.4) |
| **Full uptake (100%)** | 12.0  (5.4-17.6) | 3.3  (1.1-4.1) | 3.3  (1.1-4.1) |
| **IV. Infants of all pregnant women in Zimbabwe (HIV prevalence 16%, incidence 1%/year): proportion of annual cohort** | | | |
| **Zimbabwe 2008 (36%)** | 2.8  (2.5-3.5) | 2.5  (1.8-2.9) | 2.4  (1.7-2.9) |
| **Zimbabwe 2009 (56%)** | 2.4  (1.5-3.1) | 2.0  (1.4-2.3) | 1.8  (1.2-2.2) |
| **WHO target (80%)** | 2.0  (1.1-2.7) | 1.3  (0.9-1.5) | 1.1  (0.7-1.4) |
| **Optimal (95%) uptake** | 1.7  (0.9-2.4) | 0.9  (0.6-1.1) | 0.7  (0.4-1.0) |
| **Full uptake (100%)** | 1.6  (0.8-2.4) | 0.8  (0.5-0.9) | 0.5  (0.3-0.8) |

**Table F. Results of a model of PMTCT services in Zimbabwe: Two-year pediatric survival**

| **Uptake scenario** | **Base case model results for each PMTCT regimen (%)**  **(range, worst- to best-case MTCT risks)** | | |
| --- | --- | --- | --- |
|  | **sdNVP** | **WHO 2010 Option A** | **WHO 2010 Option B** |
| **I. Infants of all women HIV-infected before pregnancy (36% ART-eligible)** | | | |
| **Zimbabwe 2008 (36%)** | 80.1  (78.2-82.5) | 81.0  (79.7-82.9) | 81.5  (79.9-83.4) |
| **Zimbabwe 2009 (56%)** | 81.0  (79.1-83.4) | 82.4  (81.3-84.0) | 83.2  (81.7-84.7) |
| **WHO target (80%)** | 82.0  (80.0-84.3) | 83.9  (83.1-85.2) | 85.0  (83.8-86.3) |
| **Optimal (95%) uptake** | 82.8  (80.7-85.0) | 85.0  (84.4-86.0) | 86.3  (85.2-87.3) |
| **Full uptake (100%)** | 83.1  (81.1-85.3) | 85.5  (85.0-86.4) | 86.9  (85.9-87.7) |
| **II. Infants of non-ART eligible women, HIV-infected before pregnancy (CD4 > 350/µL, no WHO stage 3-4 disease)** | | | |
| **Zimbabwe 2008 (36%)** | 81.3  (79.7-83.2) | 81.9  (80.8-83.3) | 82.7  (81.2-84.2) |
| **Zimbabwe 2009 (56%)** | 82.0  (80.3-83.9) | 82.9  (82.0-84.1) | 84.1  (82.7-85.3) |
| **WHO target (80%)** | 82.8  (81.1-84.7) | 84.1  (83.4-84.9) | 85.8  (84.5-86.6) |
| **Optimal (95%) uptake** | 83.4  (81.6-85.2) | 84.8  (84.3-85.5) | 86.9  (85.6-87.5) |
| **Full uptake (100%)** | 83.6  (81.8-85.3) | 85.1  (84.8-85.7) | 87.3  (86.1-87.8) |

**Table F, continued**

| **Uptake scenario** | **Base case model results for each PMTCT regimen (%)**  **(range, worst- to best-case MTCT risks)** | | |
| --- | --- | --- | --- |
|  | **sdNVP** | **WHO 2010 Option A** | **WHO 2010 Option B** |
| **III. Infants of ART-eligible women, HIV-infected before pregnancy (CD4 ≤ 350/µL or WHO stage 3-4 disease)** | | | |
| **Zimbabwe 2008 (36%)** | 78.0  (75.6-81.3) | 79.4  (77.6-82.1) | 79.4  (77.6-82.1) |
| **Zimbabwe 2009 (56%)** | 79.4  (76.8-82.4) | 81.5  (79.9-83.7) | 81.5  (79.9-83.7) |
| **WHO target (80%)** | 80.6  (78.1-83.8) | 83.8  (82.6-85.6) | 83.8  (82.6-85.6) |
| **Optimal (95%) uptake** | 81.7  (79.2-84.7) | 85.4  (84.5-87.0) | 85.4  (84.5-87.0) |
| **Full uptake (100%)** | 82.3  (79.9-85.2) | 86.2  (85.5-87.6) | 86.2  (85.5-87.6) |
| **IV. Infants of all pregnant women in Zimbabwe (HIV prevalence 16%, incidence 1%/year)** | | | |
| **Zimbabwe 2008 (36%)** | 89.2  (89.0-89.7) | 89.4  (89.2-89.8) | 89.5  (89.2-89.9) |
| **Zimbabwe 2009 (56%)** | 89.4  (89.3-89.8) | 89.6  (89.4-89.9) | 89.7  (89.5-90.1) |
| **WHO target (80%)** | 89.6  (89.2-90.0) | 89.9  (89.7-90.1) | 90.0  (89.8-90.3) |
| **Optimal (95%) uptake** | 89.7  (89.3-90.1) | 90.0  (89.9-90.3) | 90.2  (90.1-90.5) |
| **Full uptake (100%)** | 89.7  (89.4-90.2) | 90.1  (90.0-90.3) | 90.3  (90.2-90.5) |

**Table G. Results of a model of PMTCT services in Zimbabwe: Two-year pediatric HIV-free survival**

| **Uptake scenario** | **Base case model results for each PMTCT regimen (%)**  **(range, worst- to best-case MTCT risks)** | | |
| --- | --- | --- | --- |
|  | **sdNVP** | **WHO 2010 Option A** | **WHO 2010 Option B** |
| **I. Infants of all women HIV-infected before pregnancy (36% ART-eligible)** | | | |
| **Zimbabwe 2008 (36%)** | 68.2  (62.1-75.6) | 70.6  (66.2-76.4) | 71.5  (65.9-77.6) |
| **Zimbabwe 2009 (56%)** | 70.4  (64.2-77.6) | 73.8  (70.0-78.9) | 75.2  (70.0-80.5) |
| **WHO target (80%)** | 72.8  (66.4-80.0) | 77.4  (74.2-81.8) | 79.3  (74.4-83.8) |
| **Optimal (95%) uptake** | 74.6  (68.2-81.6) | 80.1  (77.5-83.8) | 82.3  (77.7-86.2) |
| **Full uptake (100%)** | 75.5  (69.4-85.8) | 81.5  (79.7-84.7) | 83.9  (79.7-87.4) |
| **II. Infants of non-ART eligible women, HIV-infected before pregnancy (CD4 > 350/µL, no WHO stage 3-4 disease)** | | | |
| **Zimbabwe 2008 (36%)** | 71.4  (66.0-77.3) | 73.4  (70.0-77.7) | 74.8  (69.6-79.5) |
| **Zimbabwe 2009 (56%)** | 73.5  (67.5-78.9) | 75.8  (73.0-79.5) | 77.9  (72.8-81.9) |
| **WHO target (80%)** | 75.1  (69.4-80.8) | 78.6  (76.3-81.6) | 84.5  (76.5-84.5) |
| **Optimal (95%) uptake** | 76.3  (70.5-82.0) | 80.6  (78.7-82.9) | 84.0  (79.0-85.5) |
| **Full uptake (100%)** | 76.8  (70.9-82.4) | 81.5  (80.1-83.4) | 85.2  (80.1-87.6) |

**Table G, continued**

| **Uptake scenario** | **Base case model results for each PMTCT regimen (%)**  **(range, worst- to best-case MTCT risks)** | | |
| --- | --- | --- | --- |
|  | **sdNVP** | **WHO 2010 Option A** | **WHO 2010 Option B** |
| **III. Infants of ART-eligible women, HIV-infected before pregnancy (CD4 ≤ 350/µL or WHO stage 3-4 disease)** | | | |
| **Zimbabwe 2008 (36%)** | 62.7  (55.2-72.5) | 65.6  (59.5-74.2) | 65.6  (59.5-74.2) |
| **Zimbabwe 2009 (56%)** | 65.7  (58.2-75.4) | 70.2  (64.9-77.9) | 70.2  (64.9-77.9) |
| **WHO target (80%)** | 68.7  (61.1-78.5) | 75.3  (70.6-82.1) | 75.3  (70.6-82.1) |
| **Optimal (95%) uptake** | 71.4  (64.1-80.9) | 79.2  (75.4-85.2) | 79.2  (75.4-85.2) |
| **Full uptake (100%)** | 73.4  (66.8-82.3) | 81.7  (79.0-86.9) | 81.7  (79.0-86.9) |
| **IV. Infants of all pregnant women in Zimbabwe (HIV prevalence 16%, incidence 1%/year)** | | | |
| **Zimbabwe 2008 (36%)** | 87.2  (86.1-88.5) | 87.5  (86.8-88.6) | 87.7  (86.7-88.8) |
| **Zimbabwe 2009 (56%)** | 87.5  (86.5-88.8) | 88.1  (87.4-89.9) | 88.3  (87.4-90.1) |
| **WHO target (80%)** | 87.9  (86.8-89.2) | 88.6  (88.1-89.5) | 88.9  (88.1-95.0) |
| **Optimal (95%) uptake** | 88.2  (87.1-89.5) | 89.1  (88.6-89.8) | 89.4  (88.6-90.2) |
| **Full uptake (100%)** | 88.3  (87.3-89.6) | 89.3  (88.9-90.0) | 89.7  (89.0-90.4) |

**Table H. Results of additional sensitivity analyses**

| **56% Uptake scenario ^a^** | **sdNVP** | **WHO 2010 Option A** | **WHO 2010 Option B** |
| --- | --- | --- | --- |
| **Infants of women HIV-infected before pregnancy: MTCT risk (%)** | | | |
| Base-case results (for comparison; also shown in  Manuscript Table 2) ^b^ | 18.0 | 14.4 | 13.4 |
| HIV testing offered in labor to all women with unknown or previously negative HIV status (base case = 0%) ^c^ | 17.7 | 14.0 | 12.9 |
| sdNVP offered to all women identified as HIV-infected through HIV testing during labor (if previous HIV test negative or status unknown; base case = not offered) ^c^ | 15.8 | 12.2 | 11.2 |
| CD4 assay availability for women offered Option A = 0% (base case = 100% availability) | 18.0 | 15.6 | 13.4 |
| CD4 assay availability for women offered Option A = 25% ^d^ (base case = 100% availability) | 18.0 | 15.3 | 13.4 |
| CD4 result return 75% during pregnancy for women offered Option A (base case = 100% result return) | 18.0 | 14.7 | 13.4 |
| CD4 assay availability = 25% and result return = 75% for women offered Option A (base case = 100% assay availability and result return) | 18.0 | 15.4 | 13.4 |

a. Results for each regimen are shown at 56% uptake. The impact of the parameters varied in sensitivity analysis was similar at all uptake levels (36%, 56%, 80%, 95%, and 100%), except where noted.

b. HIV testing and receipt of sdNVP in labor were not available in the base-case analyses, in order to permit calibration to the total product of participation (POP, or uptake) required in each scenario. For example, in the 56% uptake scenario, if 36% of women with unknown or negative HIV status undergo testing in labor, and 80% of these women receive sdNVP, the total POP increases from 56% to 76%.

c. HIV testing in labor and offer of sdNVP in labor had less impact in high-uptake scenarios (95% and 100%) than at 36%, 56% or 80% uptake, because few women in the high-uptake scenarios had unknown HIV status at the time of labor.

d. CD4 assay availability of 25% is similar to WHO estimates of assay availability at PMTCT sites worldwide (24%) .

**REFERENCES:**
